# *Caenorhabditis elegans* DAF-16 regulates lifespan and immune responses to *Cryptococcus neoformans* and *Cryptococcus gattii* infections

**DOI:** 10.1186/s12866-022-02579-x

**Published:** 2022-06-22

**Authors:** Thitinan Kitisin, Watcharamat Muangkaew, Passanesh Sukphopetch

**Affiliations:** Department of Microbiology and Immunology, Faculty of Tropical Medicine, Mahidol University, Bangkok, Thailand

**Keywords:** *Caenorhabditis elegans*, *Cryptococcus neoformans*, *Cryptococcus gattii*, Insulin/IGF-1 signaling (IIS) pathway, DAF-16

## Abstract

**Background:**

Cryptococcosis is a life-threatening infection is primarily caused by two sibling species *Cryptococcus neoformans* and *Cryptococcus gattii.* Several virulence-related factors of these cryptococci have been widely investigated in *Caenorhabditis elegans,* representing a facile in vivo model of host–pathogen interaction. While recent studies elucidated cryptococcal virulence factors, intrinsic host factors that affect susceptibility to infections by cryptococci remain unclear and poorly investigated.

**Results:**

Here, we showed that defects in *C. elegans* insulin/insulin-like growth factor-1 (IGF-1) signaling (IIS) pathway influenced animal lifespan and mechanisms of host resistance in cryptococcal infections, which required the activation of aging regulator DAF-16/Forkhead box O transcription factor. Moreover, accumulation of lipofuscin, DAF-16 nuclear localization, and expression of superoxide dismutase (SOD-3) were elevated in *C. elegans* due to host defenses during cryptococcal infections.

**Conclusion:**

The present study demonstrated the relationship between longevity and immunity, which may provide a possibility for novel therapeutic intervention to improve host resistance against cryptococcal infections.

## Background

*Cryptococcus* species are encapsulated basidiomycetous fungi that can cause pulmonary or disseminated infections called cryptococcosis in humans and other mammals. Cryptococci can be found worldwide and are commonly involved with environmental exposures, including pigeon droppings, water, soil, or certain contaminated foods [1]. The two taxa of *Cryptococcus* species, including *Cryptococcus neoformans* species complex and *Cryptococcus gattii* species complex, are the common etiologic agents of cryptococcosis [2]. However, *C. neoformans* is generally known to cause meningitis or disseminated diseases in immunocompromised hosts. In contrast, *C. gattii* can cause pneumonia and respiratory failure in both immunocompromised and immunocompetent individuals [3]. A recent study has demonstrated that *C. neoformans* can grow faster and cause lethality faster than *C. gattii* in the murine model of Cryptococcosis [4]. Although the two *C. neoformans/C. gattii* species complex show differences in disease pathologies, the host immunological responses against these two Cryptococcal infections remain unclear and poorly determined.

Elucidating the molecular mechanisms of microbial pathogenesis in vertebrate models provide valuable insights into the pathogenic virulence factors and host responses [3]. However, severe ethical constraints, high costs, time consumption, reproducibility challenges, and physiologic and anatomic complexity in vertebrate models are often limited and cause problems in conducting the research [5]. Over the past three decades, the model of *Caenorhabditis elegans* (a soil-dwelling nematode) has been utilized to circumvent these problems and has proven to be an excellent host for studying microbial pathogenesis [6, 7]. Moreover, the killing of *C. elegans* by microbial pathogens provides a possible way to determine the pathogen virulence and host’s innate immune responses [8, 9]. In laboratory, *C. elegans* can easily feed on unicellular microbes such as a slow-growing strain of *Escherichia coli* OP50 [10]. Although *C. elegans* lack an adaptive immune system, however, innate immunity in *C. elegans* has been extensively investigated [11]. Previous studies have suggested that human pathogenic yeast, *Cryptococcus neoformans* ATCC#32045 but not *C. laurentii* or *C. kuetzingii,* can infect and kill wild-type (N2) *C. elegans* on brain heart infusion (BHI) agar [12]. Previous studies also determined the *C. neoformans* virulent factors associated with the pathogenesis in mammals and *C. elegans* [12, 13]. Many *C. neoformans* genes involving capsule growth and melanin production through the regulation of the Gα-cAMP/PKA (cAMP-dependent protein kinase A) signaling pathway that played a virulent role in mammals, were also found to enhance the killing of *C. elegans* [12]. Moreover, *C. gattii*, a closely related sibling species of *C. neoformans,* has been previously demonstrated to kill *C. elegans* [14]*.* Therefore, these studies have provided several virulent factors of cryptococcal infections upon *C. elegans* killing. However, molecular mechanisms underlying *C. elegans* responses to cryptococcal infections remain poorly determined.

Many aspects of *C. elegans* innate immunity have been showed to be involved with the longevity pathway [11]. The *C. elegans* insulin/insulin-like growth factor-1 (IGF-1) signaling (IIS) pathway controls the activity of phosphoinositide 3-kinase (PI3K)/Akt kinase cascade and regulates a Forkhead box O (FOXO) transcription factor DAF-16 and its co-mediators such as c-Jun N-terminal kinase (JNK)-1, sirtuin (SIR)-2.1, and osmotic stress resistant (OSR)-1 [15]. Collectively, the IIS pathway not only controls the lifespan of *C. elegans* but also regulates several biological activities, including development, stress responses, and innate immunity [16]. However, little is known about aging and immunity determinants that mediate host susceptibility to pathogen infections, especially cryptococci.

In this present study, we determined the correlation between longevity and immunity during *C. neoformans* and *C. gattii* infections. We demonstrated that *C. elegans* responses to both cryptococci mediated-killing were influenced by modulation of the IIS pathway, which was directly regulated by DAF-16. In addition, we provide important aspects of host innate immunity to cryptococcal infections and provide an experimental host of a simple nematode *C. elegans* for the study of cryptococcal pathogenesis and of other human pathogenic fungi.

## Results

### Mutations in the insulin/IGF-1 signaling pathway (IIS) components regulate the survival of *C. elegans* during cryptococcal infections

**Fig. 1:**
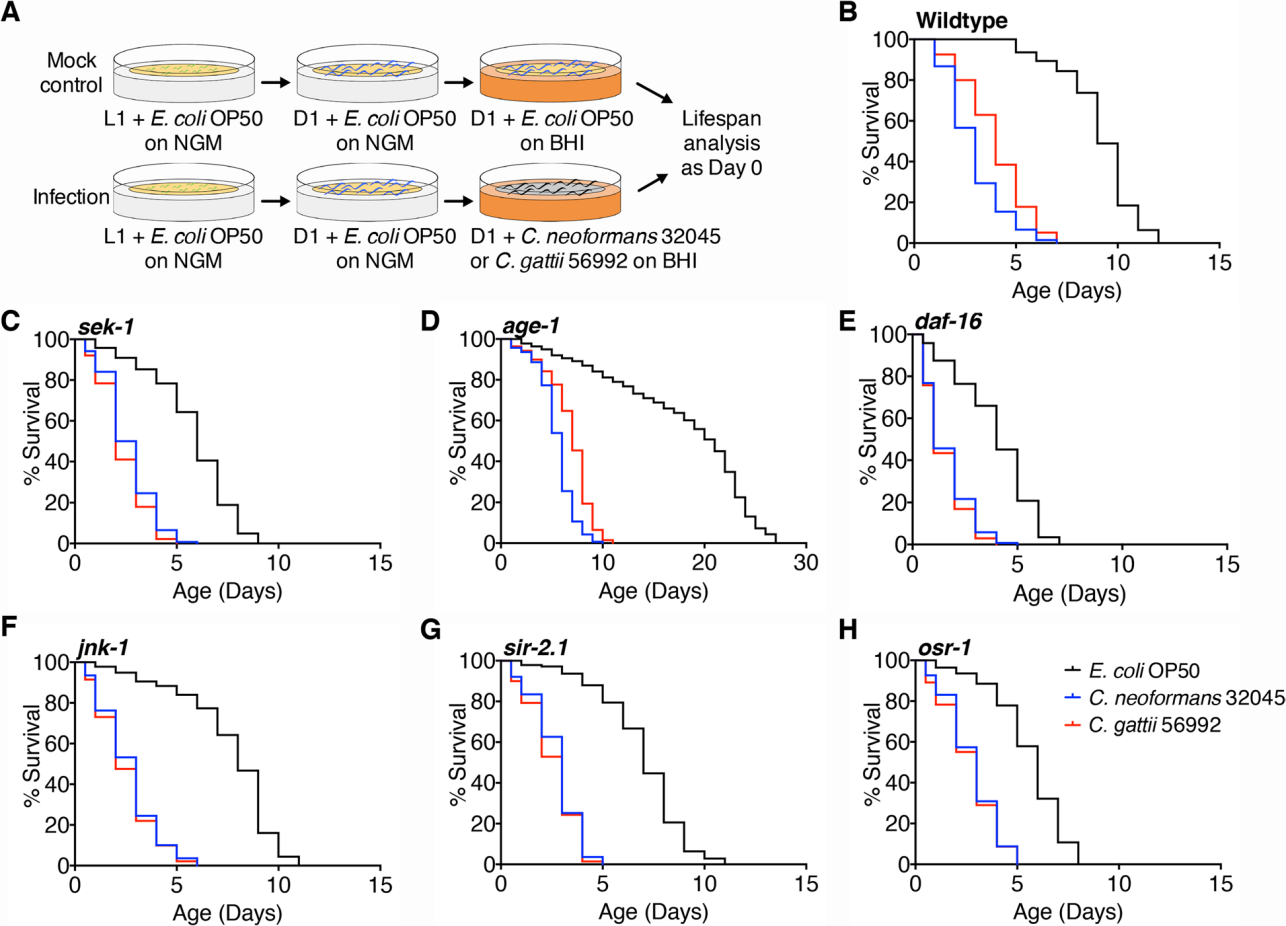
Defects in insulin/IGF-1 signaling pathway (IIS) components regulate the lifespan of *C. elegans* after feeding with *C. neoformans* and *C. gattii*. **A** a schematic diagram of the *C. elegans*-cryptococcal killing model. Wild-type and transgenic *C. elegans* at D1 adult were feeding on the lawns of *C. neoformans* 32045*, C. gattii* 56992, and *E. coli* OP50 on BHI plates at 25 °C. Percentages of survival of cryptococcal infected- **B** wild-type, **C** *sek-1(ag1)*, **D** *age-1(hx546)*, **E** *daf-16(mu86)*, **F** *jnk-1(gk7)*, **G** *sir-2.1(ok434)*, and **H** *osr-1(rm1)* strains (*n* = 50). Worms being fed with *E. coli* OP50 were served as mock-infected controls. Lifespan was analyzed by Kaplan–Meier analysis and *P*‐values were obtained from the log‐rank test. Statistical details of lifespan in each *C. elegans* strain are summarized in Table 1

In this study, we performed the solid killing assays by exposing D1 stage *C. elegans* to lawns of *C. neoformans* 32045 or *C. gattii* 56992 as a model illustrated in Fig. 1A. To prevent the possibility of the matricidal killing of *C. elegans* by cryptococcal infections, which was not associated with an infection process, we transferred the wild-type worms at the age of day 1 (D1) of adulthood from lawns of *E. coli* OP50 to lawns of *C. neoformans* or *C. gattii* on solid BHI media, which allowed worms to lay their eggs prior analysis. The lifespan was represented as days the worms survived. We demonstrated that exposing wild-type worms to lawns of two *Cryptococcus* isolates resulted in highly reproducible killing in *C. neoformans* and in *C. gattii* infected groups (Fig. 1B, *P* < 0.0001, Table 1). Interestingly, infections of *C. neoformans* significantly decreased the lifespan of wild-type worms when compared to *C. gattii* (*P* < 0.001, Table 1). These data indicated that host longevity was reduced upon cryptococcal infections and *C. neoformans* was more virulent than *C. gattii* as determined in wild-type *C. elegans*. However, more *Cryptococcus* isolates of these two species are needed to compare the virulent factors across the *Cryptococcus* species.

**Table 1:** Longevity of different *C. elegans* strains upon *C. neoformans* 32045 and *C. gattii* 56992 infections

**Strain**		**Mean**	**SD**	**SE**	**Median**	**Max**	**No.**	***P*-value**		**%**
	+ *E. coli* OP50	9.15	1.75	0.15	9.00	12.00	141			
Wild-type	+ *C. neoformans*	3.04	1.57	0.13	3.00	9.00	138	< 0.0001	****	-66.78%
	+ *C. gattii*^***###***^	4.10	1.73	0.15	4.00	9.00	139	< 0.0001	****	-55.19%
	+ *E. coli* OP50	5.79	1.99	0.17	6.00	9.00	143			
*sek-1*	+ *C. neoformans*	2.63	1.21	0.11	2.50	6.00	138	< 0.0001	****	-54.58%
	+ *C. gattii*^ns^	2.36	1.12	0.10	2.00	5.00	139	< 0.0001	****	-59.24%
	+ *E. coli* OP50	16.89	6.90	0.59	21.00	27.00	138			
*age-1*	+ *C. neoformans*	5.50	1.78	0.15	6.00	10.00	141	< 0.0001	****	-67.44%
	+ *C. gattii*^#^	6.80	2.21	0.19	7.00	11.00	139	< 0.0001	****	-59.74%
	+ *E. coli* OP50	3.97	1.76	0.15	4.00	7.00	144			
*daf-16*	+ *C. neoformans*	1.62	1.06	0.09	1.00	5.00	138	< 0.0001	****	-59.19%
	+ *C. gattii*^ns^	1.51	0.95	0.08	1.00	5.00	136	< 0.0001	****	-61.96%
	+ *E. coli* OP50	7.64	2.33	0.20	8.00	11.00	137			
*jnk-1*	+ *C. neoformans*	2.64	1.39	0.12	3.00	6.00	139	< 0.0001	****	-65.45%
	+ *C. gattii*^ns^	2.50	1.38	0.12	3.00	6.00	141	< 0.0001	****	-67.28%
	+ *E. coli* OP50	6.85	1.93	0.17	7.00	10.00	137			
*sir-2.1*	+ *C. neoformans*	2.71	1.16	0.10	3.00	5.00	139	< 0.0001	****	-60.44%
	+ *C. gattii*^ns^	2.53	1.18	0.10	3.00	5.00	140	< 0.0001	****	-63.07%
	+ *E. coli* OP50	5.57	1.71	0.14	6.00	8.00	140			
*osr-1*	+ *C. neoformans*	2.77	1.28	0.11	3.00	5.00	136	< 0.0001	****	-50.27%
	+ *C. gattii*^ns^	2.66	1.34	0.11	3.00	5.00	138	< 0.0001	****	-52.24%

*Note*: Means, standard deviation (SD), standard error (SE), median, and maximum of lifespan were shown in days. Total worms (No.) were represented as summation of worms in triplicates (with censored worms excluded). The lifespan data were analyzed using the log‐rank test and *P*‐values for each individual experiment were shown when compared to corresponding control as *P < 0.05; **P < 0.01; ***P < 0.001; ****P < 0.0001; NS, not significant (*P* > 0.05). Comparison of statistically significant differences between *C. neoformans* and *C. gattii* infected groups was indicated as ^#^*P* < 0.05; ^##^*P* < 0.01; ^###^*P* < 0.001; ^####^*P* < 0.0001; NS, not significant (*P* > 0.05). Results presented in Fig. 1B-H

It is generally known that clinical pathology caused by *C. neoformans* and *C. gattii* was different as *C. neoformans* affects immunocompromised hosts whereas *C. gattii* affects both immunocompromised and immunocompetent individuals [3]. To determine the lifespan of immunocompromised *C. elegans* during cryptococcal infections, we used a mutant *sek-1(ag1)* worms with loss-of-function of SEK-1 encoding a conserved mitogen-activated protein (MAP) kinase kinase involved in the innate immune response. The *sek-1* animals are highly susceptible to pathogen infections and relatively immunocompromised [8]. In this study, infections of *C. neoformans* and *C. gattii* in *sek-1(ag1)* worms decreased mean survival relative to the mock-infected control worms (Fig. 1C,* P* < 0.0001, Table 1). However, infections of *C. neoformans* did not significantly decrease the lifespan of *sek-1* worms when compared to *C. gattii* (*P* > 0.001, Table 1). These data suggested that other immunological mechanisms may involve and play a crosstalk role in the MAP kinase pathway during host response against infections.

In *C. elegans*, longevity is governed by the IIS pathway through the FOXO-family transcription factor DAF-16 as such a strong loss-of-function mutation in *daf-16* suppressed the long-lived phenotype of *age-1* mutant [17]. Moreover, previous studies have suggested that IIS/DAF-16 pathway and p38 MAP kinase pathway via SEK-1 may intersect during pathogenic infections [18, 19]. Given the evidence that activation of innate immune response by IIS pathway protects *C. elegans* from pathogen infections [20]. We asked whether the survival rates of loss-of-function mutants in IIS pathways such as *age-1(hx546)*, *daf-16(mu86)*, *jnk-1(gk7)*, *sir-2.1(ok434)*, *osr-1(rm1)* strains, differ between *C. neoformans* and *C. gattii* infections. In this study, infections of *C. neoformans* and *C. gattii* in *age-1(hx546)* worms decreased mean survival relative to the mock-infected control worms (Fig. 1D, *P *< 0.0001, Table 1). Moreover, infections of *C. neoformans* significantly decreased the survival of *age-1* worms when compared to *C. gattii* (*P* < 0.001, Table 1). Infections of *C. neoformans* and *C. gattii* in worms with *daf-16(mu86)* mutation decreased mean survival relative to the mock-infected control worms (Fig. 1E, *P* < 0.0001, Table 1). In *C. elegans*, several DAF-16 mediators, including JNK-1 [21], SIR-2.1 [22], OSR-1 [23], are involved in the longevity pathway as well as in stress responses and microbial infections. Similarity, infections of *C. neoformans* and *C. gattii* in worms with mutations in *jnk-1(gk7)*, *sir-2.1(ok434)*, and *osr-1(rm1)* also reduced the mean lifespan compared with the mock-infected controls (Fig. 1F-H, *P* < 0.0001, Table 1). The significant killing of *C. neoformans* than *C. gattii* in both wild-type and *age-1* (long-lived) worms demonstrated that *C. neoformans* is a more notorious pathogen than *C. gattii.* Our study suggested that longevity and innate immunity may coordinate at the molecular level in response to protect *C. elegans* from cryptococcal infections.

### Mutations in IIS/DAF-16 components of *C. elegans* regulate the host susceptibility to cryptococcal infections

**Fig. 2:**
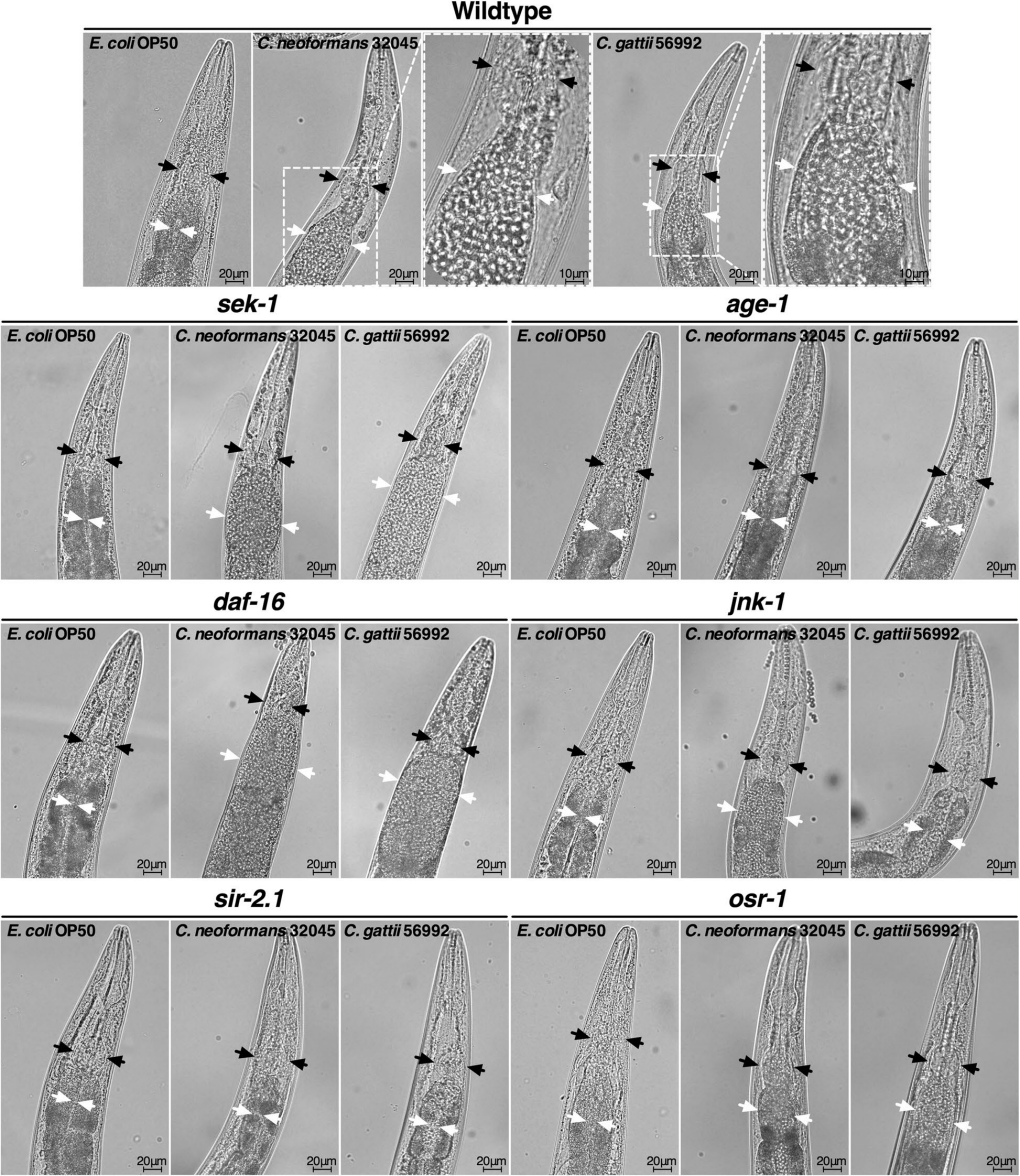
Interaction of *C. elegans* with *C. neoformans* and *C. gattii*. Survival of cryptococcal yeast cells that passed through the nematode pharyngeal grinder was seen at the distal area of the abnormally distended gastrointestinal tract of wild-type, *sek-1(ag1)*, *daf-16(mu86)*, *jnk-1(gk7)*, and *osr-1(rm1)* but not *age-1(hx546)* and *sir-2.1(ok434)* strains. Black arrows indicate the nematode pharyngeal grinder organ. White arrows indicate the intestinal lumen of the nematode. Worms were photographed alive after infecting with *C. neoformans* 32045 and *C. gattii* 56992 on BHI plates for 48 h at 25 °C (*n* = 30). Scale bar = 20 µm and 10 µm

First, we performed the microscopic evaluation of wild-type nematodes exposed to *C. neoformans* 32045 or *C. gattii* 56992 and compared them to standard food source *E. coli* OP50 [6]. We observed that both *C. neoformans* and *C. gattii* yeast cells could accumulate and are clearly seen inside an abnormally distended gastrointestinal tract after 24 h post inoculation (Fig. 2). The yeast cells were mostly accumulated at the abnormally distended intestine distally directed to the pharyngeal grinder (an organ that ruptures ingested materials during feeding) of the worms. Moreover, the yeast cells can be observed throughout the gastrointestinal tract of the worms. A previous study has suggested that the *C. neoformans* yeast cells that accumulate inside the nematode gastrointestinal tract are due to the collective ingestion rather than proliferation of the yeast cells [12]. Thus, the abnormally distended intestine may be the direct cause of death from the killing of cryptococci.

The previous study has attempted to determine *C. elegans* host factors that regulate susceptibility to *C. neoformans* infections [24]. The study demonstrated that male *C. elegans* are more resistant to *C. neoformans* killing than hermaphrodites, which is directly dictated by DAF-16. Thus, we explored and elucidated more about the role of IIS/DAF-16 components in *C. elegans* in response to cryptococci-mediated killing. We exposed worms that exhibited loss-of-function in IIS pathways mutants to the lawns of *C. neoformans* 32045 or *C. gattii* 56992. After 24 h post inoculation, we observed that both *C. neoformans* and *C. gattii* yeast cells accumulated in the gastrointestinal tract of *sek-1(ag1)*, *daf-16(mu86)*, *jnk-1(gk7)*, and *osr-1(rm1)* mutants but not in *age-1(hx546)* and *sir-2.1(ok434)* mutants (Fig. 2). Interestingly, cryptococcal infected *sek-1(ag1)* and *daf-16(mu86)* mutants exhibited highly severe intestinal distension than other transgenic nematodes. Nevertheless, although no yeast cells accumulated in the gastrointestinal tract of *age-1(hx546)* and *sir-2.1(ok434)* nematodes, their lifespans were still markedly shorter than the mean normal survival time when fed with a standard diet of *E. coli* OP50. Our results suggested the possibility of other separate mechanisms that regulate both immunity and longevity in *C. elegans*.

### Expressions of intestinal lipofuscin, DAF-16 nuclear localization, SOD-3, and NLP-29 in *C. elegans* responses to cryptococcal infections

**Fig. 3:**
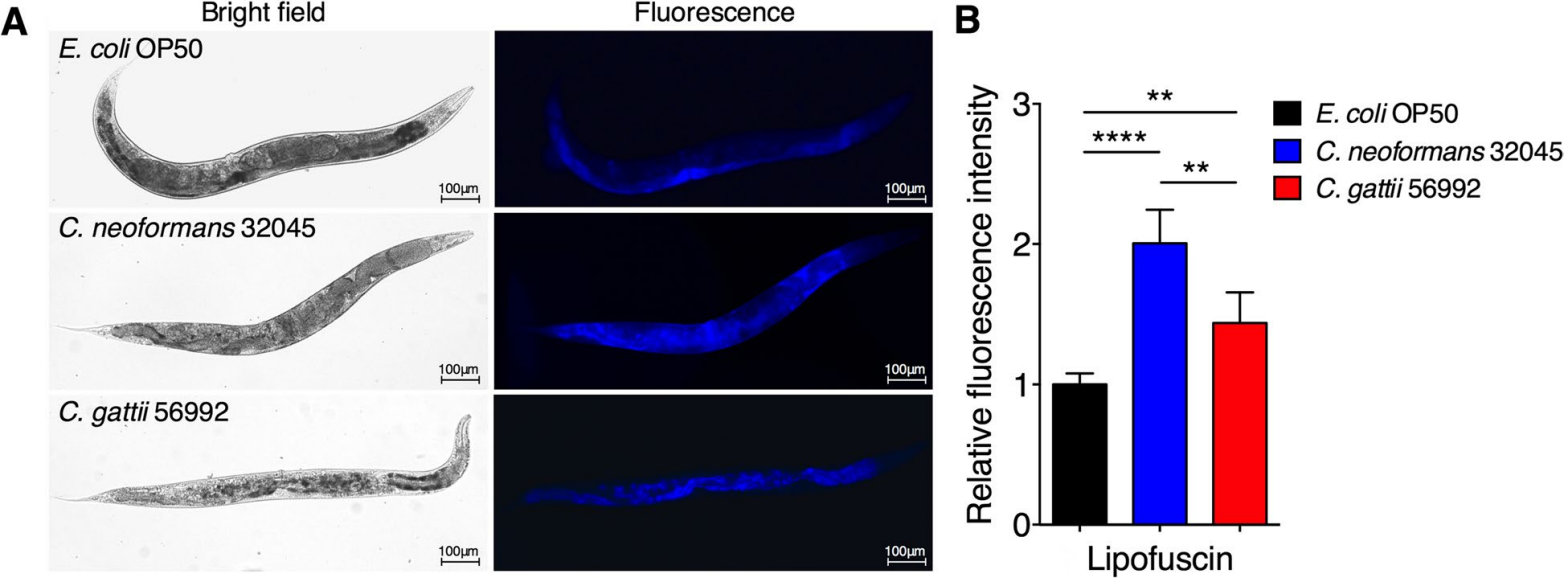
Elevation of lipofuscin accumulation in *C. elegans* intestines after feeding with *C. neoformans* and *C. gattii*. **A** Representative images of lipofuscin autofluorescence of *E. coli* OP50-, *C. neoformans*-*,* and *C. gattii*-infected wild-type nematodes. All worms were exposed to *E. coli* OP50, *C. neoformans* 32045*,* and *C. gattii* 56992 on BHI plates for 48 h at 25 °C, then imaged. **B** Relative lipofuscin autofluorescence intensity was evaluated by using ImageJ software, and the mean lipofuscin autofluorescence of mock-infected nematodes was set as 1. Data were represented in mean ± SD (*n* = 30). *P < 0.05; **P < 0.01; ***P < 0.001; ****P < 0.0001; not significant (*P* > 0.05). Scale bar = 100 µm

Although pathogen virulence of cryptococci has been extensively studied [10, 12, 13], little is known about host determinants involving responses in immunity, longevity, and cellular fitness during cryptococcal infections. To determine whether cryptococci influence the health span of *C. elegans*, we analyzed the lipofuscin (an aging pigment) of nematode [25] after *C. neoformans* 32045 or *C. gattii* 56992 infections. The intestinal autofluorescence of lipofuscin in *C. neoformans* or *C. gattii* infected wild-type worms was measured after 48 h post inoculation on BHI plates. As shown in Fig. 3, the level of lipofuscin in *C. neoformans* infected group was significantly increased from 1.00 ± 0.07 folds to 2.00 ± 0.24 (*P* < 0.0001) and higher than *C. gattii* infected group (1.43 ± 0.21, *P* < 0.01).

**Fig. 4:**
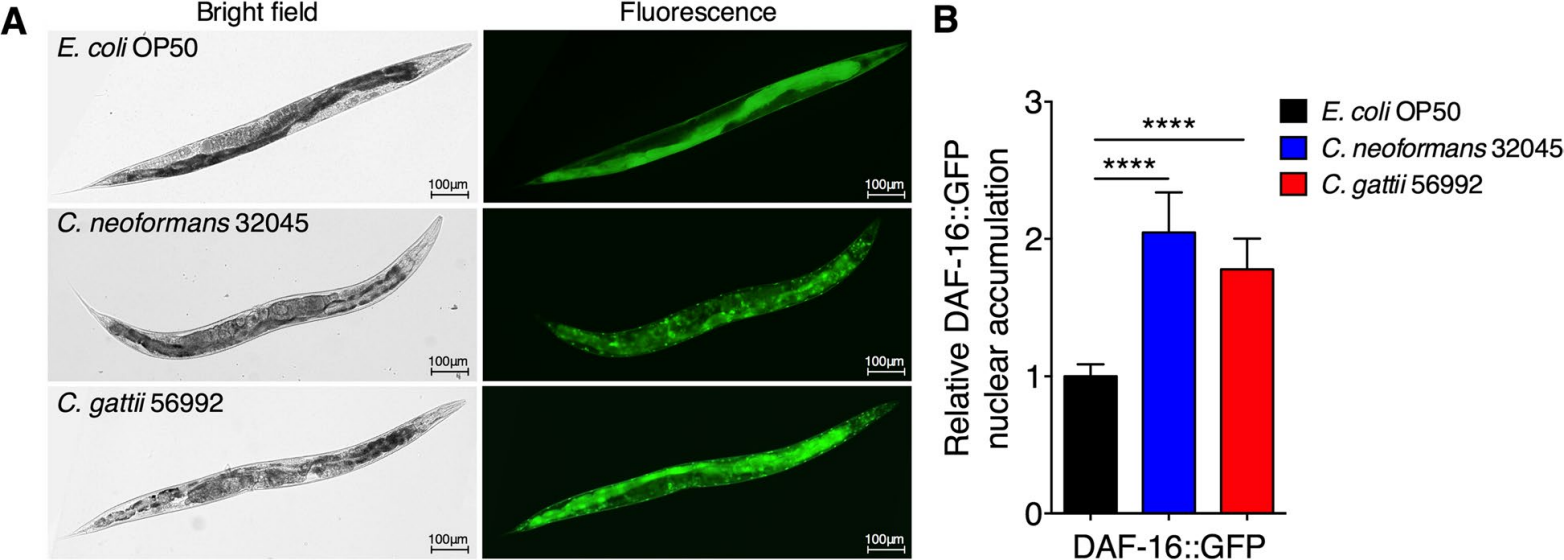
Induction of DAF‐16::GFP nuclear localization in *C. elegans* after feeding with *C. neoformans* and *C. gattii*. **A** Representative image of DAF-16 nuclear localization of *E. coli* OP50-, *C. neoformans*-*,* and *C. gattii*-infected *daf-16p::GFP* (*zIs356*) nematodes. All worms were exposed to *E. coli* OP50, *C. neoformans* 32045*,* and *C. gattii* 56992 on BHI plates for 48 h at 25 °C, then imaged. **B** Relative DAF-16::GFP nuclear accumulation was evaluated by using ImageJ software, and the mean DAF-16::GFP nuclear accumulation of mock-infected nematodes was set as 1. Data were represented in mean ± SD (*n* = 30). *P < 0.05; **P < 0.01; ***P < 0.001; ****P < 0.0001; not significant (*P* > 0.05). Scale bar = 100 µm

In the present study, our results suggested that cryptococcal infections in worms with deficient transcription factor DAF-16 (*daf-16(mu86)*) exhibited the shortest lifespan and DAF-16 may play a central role in host determinants against cryptococcal infections. To further elaborate this hypothesis, we examined the nuclear localization of DAF-16 using transgenic worms *zIs356 (daf-16::gfp)* expressing DAF-16 linked to GFP [26]. First, we examined that no DAF-16::GFP nuclear localization in worms under well-fed *E. coli* OP50 conditions, which served as mock-infected controls (Fig. 4). We observed the accumulation of *C. neoformans* 32045 and *C. gattii* 56992 yeast cells inside the *zIs356* nematode gastrointestinal tract after 24 h post inoculation (Fig. 4A). Interestingly, both *C. neoformans* and *C. gattii* infections significantly induced DAF-16 nuclear translocation from 1.00 ± 0.08 folds to 2.05 ± 0.29 (*P* < 0.0001) and to 1.78 ± 0.23 folds (*P* < 0.0001) (Fig. 4B).

**Fig. 5:**
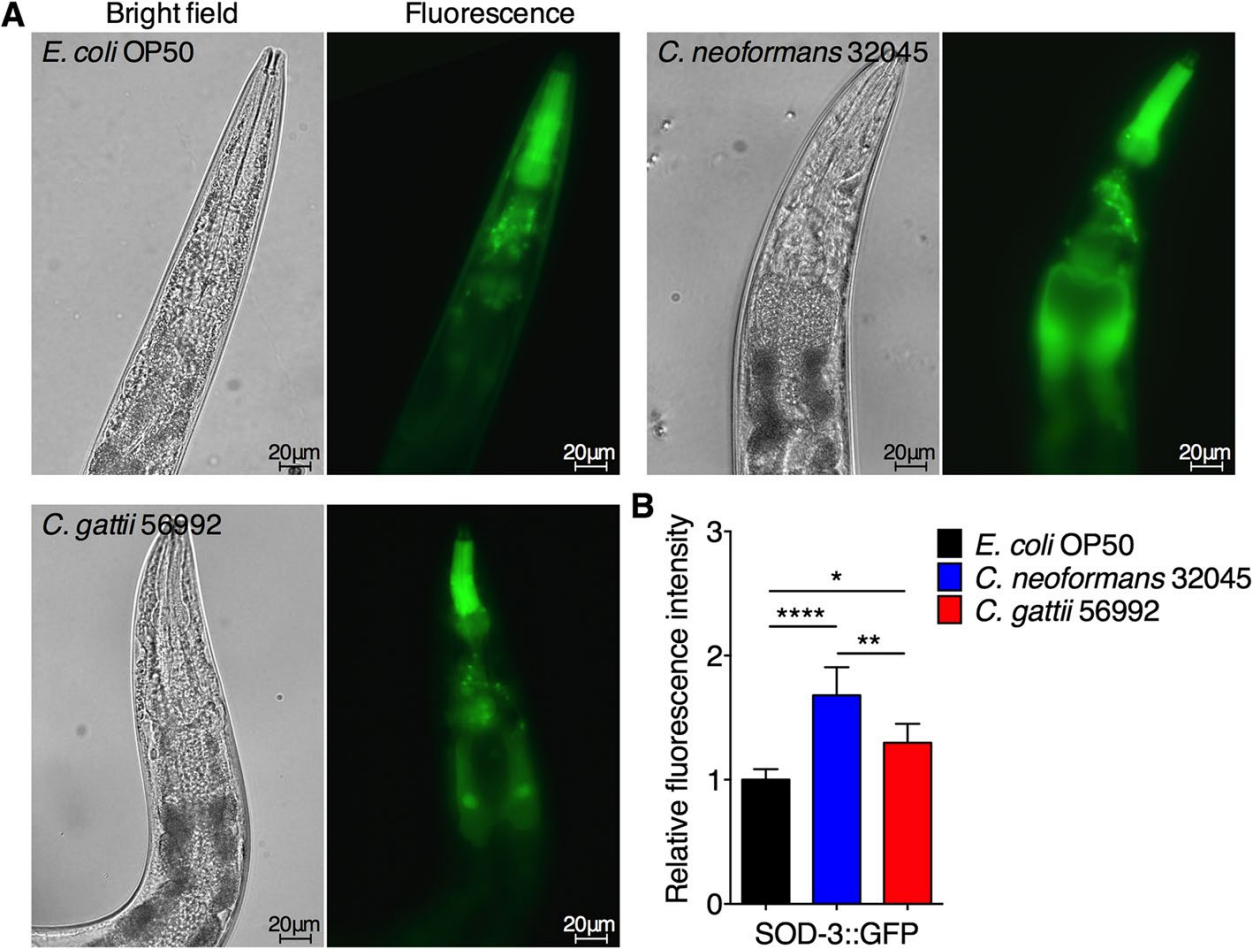
Expression of SOD-3::GFP in *C. elegans* head region after feeding with *C. neoformans* and *C. gattii*. **A** Representative image of SOD-3 expression at the head region of *E. coli* OP50-, *C. neoformans*-*,* and *C. gattii*-infected *sod-3p::GFP* (*muIs84*) nematodes. All worms were exposed to *E. coli* OP50, *C. neoformans* 32045*,* and *C. gattii* 56992 on BHI plates for 48 h at 25 °C, then imaged. **B** Relative SOD-3::GFP expression was evaluated by using ImageJ software, and the mean SOD-3::GFP expression of mock-infected nematodes was set as 1. Data were represented in mean ± SD (*n* = 30). *P < 0.05; **P < 0.01; ***P < 0.001; ****P < 0.0001; not significant (*P* > 0.05). Scale bar = 20 µm

In *C. elegans*, activation of DAF-16 has been shown to induce a DAF-16-dependent-superoxide dismutase (SOD)-3 expression during *Enterococcus faecalis* infections [27]. To determine the SOD-3 expression in the nematode intestinal cells during cryptococci infections, we used the transgenic worms *muIs84 (sod-3::gfp)* expressing a SOD-3 linked to GFP primarily in the head-, tail-neurons, and around vulva under well-fed *E. coli* OP50 condition [28]. We observed the accumulation of yeast cells inside the *muIs84* nematode gastrointestinal tract after 24 h post inoculation (Fig. 5A). Expressions of SOD-3::GFP in the head region of worm infected with both *C. neoformans* (1.68 ± 0.23 folds, *P* < 0.0001) and *C. gattii* (1.30 ± 0.15 folds, *P* < 0.05) were significantly higher than controls (1.00 ± 0.08 folds) (Fig. 5B). Moreover, the expression level of SOD-3::GFP in *C. neoformans* infected group was significantly higher than *C. gattii* infected group (*P* < 0.01).

**Fig. 6:**
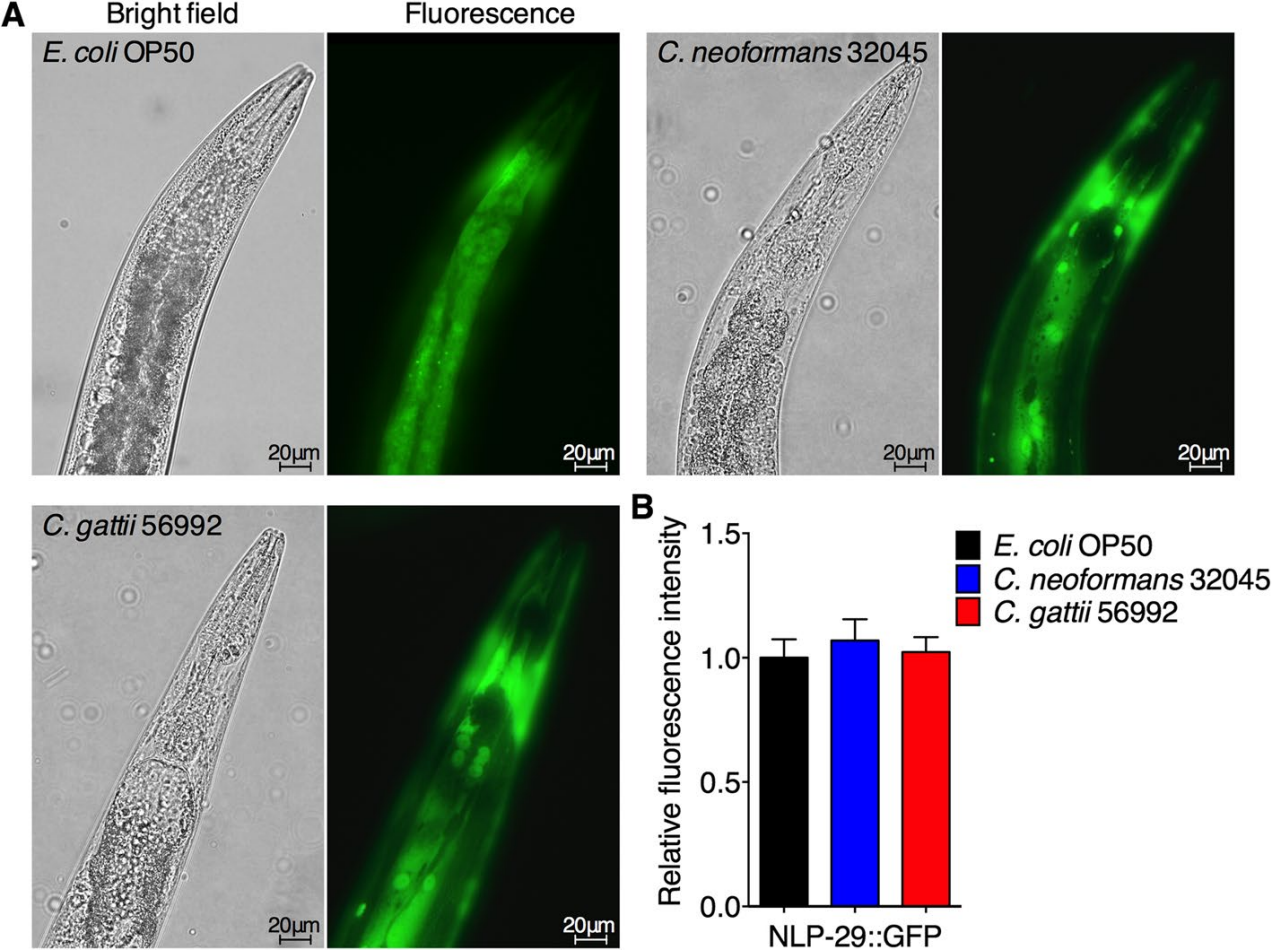
Expression of NLP-29::GFP in *C. elegans* after feeding with *C. neoformans* and *C. gattii*. **A** Representative image of NLP-29 expression at the head region of *E. coli* OP50-, *C. neoformans*-*,* and *C. gattii*-infected *nlp-29p::gfp* (*fasn-1(fr8);frIs7*) nematodes. All worms were exposed to *E. coli* OP50, *C. neoformans* 32045*,* and *C. gattii* 56992 on BHI plates for 48 h at 25 °C, then imaged. **B** Relative NLP-29::GFP expression was evaluated by using ImageJ software, and the mean NLP-29::GFP expression of mock-infected nematodes was set as 1. Data were represented in mean ± SD (*n* = 30). *P < 0.05; **P < 0.01; ***P < 0.001; ****P < 0.0001; not significant (*P* > 0.05). Scale bar = 20 µm

The previous finding demonstrated that *C. elegans* produces antimicrobial peptides (AMPs) as an innate defense mechanism against nematophagous fungi, *Drechmeria coniospora,* which was regulated by *nlp-29* gene via the DAF-16 pathway [29, 30]. To determine the expression of antimicrobial peptides NLP-29 in the nematode intestinal cells during cryptococci infections, we used the transgenic worms *fasn-1(fr8);frIs7 (nlp-29::gfp)* that constitutively express AMPs [31]. We observed the accumulation of yeast cells inside the worms *fasn-1(fr8);frIs7* nematode gastrointestinal tract after 24 h post inoculation (Fig. 6A). Expressions of NLP-29::GFP in the intestinal region of worm infected with both *C. neoformans* (1.07 ± 0.08 folds, *P* > 0.05) and *C. gattii* (1.02 ± 0.06 folds, *P* > 0.05) were insignificant differences when compared to controls (1.00 ± 0.07 folds) (Fig. 6B). Taken together, these data suggested that infections of cryptococci worsen host fitness by inducing aging pigment, which activates antifungal immune- and stress-responses. Further study is required to elucidate an additional factor that controls DAF-16 activity on immunity and longevity during fungal infections.

## Discussion

In this paper, we utilized a round worm of *C. elegans* as an experimental host–pathogen system to determine pathological and physiological responses against cryptococcal infections*.* Despite the fact that cryptococcal yeast cells are relatively large when compared to bacterial cells [12], we found that wild-type (N2) *C. elegans* can ingest both *C. neoformans* 32045 and *C. gattii* 56992. In addition, those cryptococci killed the *C. elegans* after feeding. Thus, the *C. elegans*/cryptococci infection model can be used to determine host responses against infections*.* Further study is required to validate the cryptococcal pathogenesis in mammals that was identified in the *C. elegans* model.

Several established experiments using *C. elegans* as a host organism to study microbial pathogenesis have faced an internal hatching or matricidal hatching, causing premature death of worms [32]. To succumb to this phenomenon, transgenic *fer-15(b26);fem-1(hc17) C. elegans* that sterilization was induced by temperature or by adding 5-fluoro-2ʹ-deoxyuridine (FUdR) into the worm culture to prevent the progeny from hatching are widely used [33–35]. However, utilization of temperature-induced sterile worms or adding FUdR may not provide a proper strategy to study the intrinsic host determinants in other transgenic *C. elegans.* Therefore, in our study, we strategically exposed *C. elegans* at day 1 of adulthood (D1) rather than the fourth larval stage (L4), allowing worms to expel their eggs prior to infections. Thus, we succeeded in performing *C. elegans-*cryptococci killing assay without matricidal hatching without the addition of FUdR.

The effectiveness of host defenses against pathogenic infections is a ubiquitous challenge and contributes to an important aspect of life. Both host tolerance and resistance to pathogens are highly regulated through a strong evolutionary selection [36]. In *C. elegans*, the evolutionarily conserved longevity determinants are highly regulated in the insulin/IGF-1 signaling (IIS) and a Forkhead box O (FOXO) transcription factor DAF-16 pathway, which is shown to play a central role in determining *C. elegans* aging. Moreover, many molecular determinants in longevity also regulate the ability of *C. elegans* resistance to the pathogen (for review, see [15, 37–39]). A previous study also demonstrated that immunocompromised *C. elegans* with a deficiency in *sek-1* are highly susceptible to *P. aeruginosa* [8] and *Candida albicans* infections [40]. Moreover, previous study has suggested that defects in IIS/DAF-16 and p38 MAP kinase pathways renders *C. elegans* more susceptible to *P. aeruginosa* infections [18]. In the present study, highly severe intestinal distension caused by infections of cryptococci was found most in infected *sek-1(ag1)* and *daf-16(mu86)* mutants than in other transgenic nematodes. These observations have led to the suggestion that defects in both longevity (*daf-16*) and immunity (*sek-1*) may be responsible for increased susceptibility to cryptococcal infections. Previous study has suggested that DAF-16 plays a central role in regulating oxidative stress responses leading to activation of the p38-related SEK-1 (MAPKK) pathway, which in turn activates an innate immune response in *C. elegans* [19]. Thus, our study suggested that host immunological responses are partially under the molecular pathways regulating host longevity.

An increase in resistance and longevity occurs when partial loss-of-function of the phosphatidylinositol-3-OH kinase (PI(3)K)-encoding gene *age-1*, which relieves the suppression of DAF-16 [41, 42]. Previously study has shown that long-lived *age-1* mutant nematodes exhibited a significantly higher resistance to infection by *Enterococcus faecalis, Staphylococcus aureus,* and *Pseudomonas aeruginosa* than wild-type worms [43]. However, the pathogenic resistant phenomena were suppressed in *daf-16* loss-of-function mutants [43], suggesting that DAF-16 plays an important role in host defense against infections. In our present study, we found that host longevity was reduced upon both *C. neoformans* 32045 and *C. gattii* 56992 infections in age*-1* and *daf-16* mutants (IIS determinants experimented in our study). Infected *C. elegans* by cryptococci was presented as the accumulation of yeast cells within the gastrointestinal tract and apparently killed the host. Thus, our results suggested that *C. neoformans* 32045 and *C. gattii* 56992 are not nutritious food sources for *C. elegans*. It is possible that the accumulation of cryptococci yeast cells in the *C. elegans* intestine and an abnormal distention of the intestine is sufficient for killing. However, there was no yeast cells accumulation in the infected *age-1* mutants (Fig. 2), but the nematode lifespan was still reduced (Fig. 1D). Thus, it is possible that other mechanisms such as toxin-mediated killing, may involve as observed in the study of *P. aeruginosa* killing of *C. elegans* [44, 45].

In addition to the role of DAF-16 during infections of cryptococci, we further determined DAF-16 mediators that also regulate the lifespan of *C. elegans*, including stress resistance by c-Jun N-terminal kinase (JNK) [46], calorie restriction by protein deacetylase (SIR) [47], superoxide dismutase (SOD), and osmotic stress resistant (OSR) [46]. In the present study, we found that infections of *C. neoformans* or *C. gattii* in loss-of-function *jnk-1*, *sir-2.1*, and *osr-1* mutants shortened the lifespan of animals as seen in *sek-1* mutants. Taken together, our study suggested that several intrinsic determinants in IIS/DAF-16 pathway are integrated with responses against cryptococcal infections. Therefore, infections by cryptococci, either direct or indirect, can impact host longevity and immunity.

As shortened host longevity upon cryptococcal infections, several host fitnesses were interrupted. Our recent study showed that elevation of intracellular oxidative stress could accelerates aging process and induces the accumulation of lipofuscin in *C. elegans* [48]. Moreover, previous study demonstrated an increased expression of SOD-3 in *E. faecalis* infected *C. elegans*, which was dependently associated with DAF-16 [27]. In our present study, we found that infection of cryptococci induced intestinal accumulation of lipofuscin or aging pigments in infected animals. Infections of cryptococci also induced the nuclear localization of DAF-16::GFP and elevated SOD-3::GFP expressions in the infected *C. elegans*. Thus, our findings suggested that an increase in physical injury and oxidative stress may cause by cryptococcal infections, which are largely dependent on DAF-16 [49]. Previous study has demonstrated that infections by *Drechmeria coniospora*, an obligate fungal pathogen of *C. elegans* induced the expressions of immune effector *nlp-29* encoding for antimicrobial peptides (AMPs) [29], which was regulated by the p38 MAPK pathway in the *C. elegans* epidermis [50]. In the present study, we found that NLP-29::GFP levels of constitutively active *nlp-29::gfp* mutant nematodes did not significantly change in cryptococcal infected groups and controls. Our study suggested that host-epidermal response to damage may indirectly enhance innate immunity during cryptococcal infections in *C. elegans* and expressions of AMPs may not be sufficient to protect *C. elegans* from the immediate killing of cryptococcal infections. Moreover, our study speculated that an increase in DAF-16 activity, as well as its ortholog(s) or its downstream effectors, may play a role in the regulation of longevity and cryptococcal resistance.

Previously, our laboratory has shown that *C. elegans* can be used to determine the host determinants to better understanding the pathogenesis of *C. albicans* infections [51]. Interestingly, we found that dysfunctions in the IIS/DAF-16 pathway affect the hyphal formation in the *C. albicans*-infected *C. elegans.* The results reported here suggest that a similar approach can be used to determine *C. elegans* responses against cryptococcal pathogenesis. It is important to note that the IIS/DAF-16 pathway is highly conserved to regulate aging and longevity across diverse species from invertebrates to mammals [38]. Therefore, our study suggested that the IIS/DAF-16 pathway may mainly act as evolutionarily conserved signaling pathway of the innate immune response to microbial infections in *C. elegans*.

## Conclusion

In summary, we show here that *C. elegans* can be utilized as a model host for the study of host responses against the lethal fungal pathogen, *C. neoformans* and *C. gattii*. The interaction of *C. elegans* with the cryptococci involved several intrinsic host determinants in IIS/DAF-16 pathway. Moreover, resistance to cryptococcal infections in *C. elegans* is largely dependent on DAF-16, which promotes host longevity and immunity. Thus, our study demonstrated that by using *C. elegans* as an in vivo model, we could explore the intrinsic link between longevity and immunity at the molecular level in which alteration of this evolutionarily conserved longevity pathway may deepen our understanding of the host–pathogen interaction. Further identification of these functional details in *C. elegans* may lead to the discovery of novel therapies, which can be expanded and applied to human diseases in the near future.

## Materials and methods

### Fungal strains and growth conditions

*C. neoformans* 32045 and *C. gattii* 56992 strains were obtained from the American Type Culture Collection (ATCC). Stock cultures were stored at 25% glycerol at − 80 °C until use and were maintained on Sabouraud dextrose agar (SDA, Oxoid, Hampshire, UK) at 37 °C for 2 days.

### In vivo experimental model of fungal infections

*Caenorhabditis elegans* strains used in this study were Bristol N2 (wild-type), AU1 *sek-1(ag1)*, TJ1052 *age-1(hx546)*, CF1038 *daf-16(mu86)*, VC8 *jnk-1(gk7)*, VC199 *sir-2.1(ok434)*, AM1 *osr-1(rm1)*, TJ356 *zIs356 [daf-16p::daf-16a/b::GFP* + *rol-6(su1006)]*, CF1553 *muIs84 [(pAD76) sod-3p::GFP* + *rol-6(su1006)]*, and IG348 *fasn-1(fr8);frIs7[nlp-29p::gfp, col-12p::DsRed]*. All worms were age‐synchronized using hypochlorite treatment of gravid hermaphrodites (500 μl of 1 M NaOH, 600 μl of 1 M NaClO, and 3.9 ml of distilled water) as previously described [48]. The first stage (L1) larvae were transferred into fresh nematode growth medium (NGM) plates and fed on live‐*Escherichia coli* strain OP50 at 20 °C. Bacteria were grown in Luria Broth (LB, BD).

For *C. elegans-Cryptococcus* infection, yeast cells were collected from SDA plates, washed twice with M9 buffer, and adjusted to 5 × 10^7^ cells/ml in M9 buffer. Freshly lawn of *C. neoformans* 32045 and *C. gattii* 56992 were prepared by spreading 100 µl of the yeast cells (5 × 10^7^ cells/ml) on the NGM plates and grown overnight at 25 °C. To perform an infection, 50 *C. elegans* animals at the day 1 (D1) of adulthood were transferred from a lawn of *E. coli* OP50 on NGM to the prepared yeast cells on brain heart infusion agar (BHI, Difco) and incubated at 25 °C for 24 h [12]. Nematode survival was examined under a stereomicroscope and counted every 24 h intervals until all the worms died. When the worms did not respond to touch with a platinum wire pick or did not show pharyngeal pumping movement, they were considered dead. In addition, worms were transferred to new plates every 48 h to prevent the presence of progeny. Worms that suffered from developmental defects or crawled off the plate were eliminated from the analysis. Worms that fed with *E. coli* OP50 were used as mock-infected controls. Total worms were counted as a summation of worms in triplicates, excluding the censored worms. The number of surviving and dead worms was statistically analyzed. Each experimental condition was performed in triplicate.

### Microscopic studies

Yeast infected and control of thirty synchronized D1 nematodes raised on BHI agar plates for 48 h were placed on 2% agarose pad and were immobilized with 5 μl of 30 mM sodium azide (NaN_3_) in M9 buffer. Worms were photographed using a Zeiss Axio Imager fluorescence microscope (ZEISS, Germany) using an LED source, and DAPI or GFP filters.

### Intestinal lipofuscin accumulation, DAF-16 nuclear translocation, SOD-3 and NLP-29 expressions in *C. elegans*

Yeast infected and control of wild-type N2 worms were used to determine the level of intestinal lipofuscin [48]. After 48 h post-infection, the level of lipofuscin intestinal autofluorescence was analyzed through a DAPI filter and quantified by ImageJ (National Institutes of Health, Bethesda, MD, USA) in each worm’s intestine, excluding head and tail regions. Yeast infected and control of *C. elegans* strain TJ356 *zIs356 [daf-16p::daf-16a/b::GFP* + *rol-6(su1006)]*, CF1553 *muIs84 [(pAD76) sod-3p::GFP* + *rol-6(su1006)]*, and IG348 *fasn-1(fr8); frIs7[nlp-29p::gfp, col-12p::DsRed]*, were used to determine the nuclear translocation of DAF-16, the expression of SOD-3 or NLP-29, respectively. The GFP intensity of DAF-16 nuclear translocation, SOD-3, and NLP-29 expressions in animals was photographed and quantified by ImageJ.

### Data analysis

Statistical analyses were determined using GraphPad Prism software (GraphPad Software, Inc., San Diego, California, USA). Data were presented as mean ± standard deviation (SD). All the experiments were performed in triplicate. Estimation of lifespan differences was performed with Kaplan–Meier lifespan analysis, and *P*-values were calculated using the log-rank test. One way or two-way ANOVA was conducted to test the differences. Levels of significance were indicated as *P < 0.05; **P < 0.01; ***P < 0.001; ****P < 0.0001; NS, not significant (*P* > 0.05).

## Data Availability

The datasets generated and/or analyzed during the current study are available from the corresponding author on reasonable request.

## Abbreviations

*C. elegans*: *Caenorhabditis elegans*
*C. neoformans*: *Cryptococcus neoformans*
*C. gattii*: *Cryptococcus gattii*
*E. coli*: *Escherichia coli*
IIS: Insulin/Insulin-like Growth Factor-1 (IGF-1) Signaling Pathway
SOD: Superoxide Dismutase
PI3K: Phosphoinositide 3-Kinase
FOXO: Forkhead box O
JNK: c-Jun N-terminal Kinase
SIR: Sirtuin
OSR: Osmotic Stress Resistant
MAP: Mitogen-Activated Protein
AMPs: Antimicrobial Peptides
FUdR: 5-Fluoro-2ʹ-deoxyuridine
